# Segmental and Tandem Duplications Driving the Recent *NBS-LRR* Gene Expansion in the Asparagus Genome

**DOI:** 10.3390/genes9120568

**Published:** 2018-11-23

**Authors:** Jose V. Die, Patricia Castro, Teresa Millán, Juan Gil

**Affiliations:** Department of Genetics, ETSIAM, University of Córdoba, 14071 Córdoba, Spain; patricia.castro@uco.es (P.C.); teresa.millan@uco.es (T.M.); juan.gil@uco.es (J.G.)

**Keywords:** asparagus, coiled coil, disease resistance, duplications, nucleotide binding site, plant defense, promoter, regulatory elements

## Abstract

Garden asparagus is an important horticultural plant worldwide. It is, however, susceptible to a variety of diseases, which can affect the potential yield, spear quality, and lifespan of production fields. Screening studies have identified resistant germplasm. The genetic resistance is usually complex, and the genes underlying that resistance are still unknown. Most often, disease resistance is determined by resistance genes (*R*). The most predominant *R*-genes contain nucleotide binding site and leucine-rich repeat (NBS-LRR) domains. Using bioinformatics and data mining approaches, we identified and characterized 68 NBS predicted proteins encoded by 49 different loci in the asparagus genome. The NBS-encoding genes were grouped into seven distinct classes based on their domain architecture. The *NBS* genes are unevenly distributed through the genome and nearly 50% of the genes are present in clusters. Chromosome 6 is significantly NBS-enriched and one single cluster hosts 10% of the genes. Phylogenetic analysis points to their diversification into three families during their evolution. Recent duplications are likely to have dominated the *NBS* expansion with both tandem genes and duplication events across multiple chromosomes. Transcriptome sequencing data provided evidence for their transcription and tissue-specific expression. The total number of *cis*-regulatory elements as well as their relative positions within the NBS promoters suggests a complex transcriptional network regulating defense responses. Our study provides a strong groundwork for the isolation of candidate *R*-genes in garden asparagus.

## 1. Introduction

Garden asparagus (*Asparagus officinalis* L.) is the most economically important species of the *Asparagus* genus and the only one cultivated worldwide as a vegetable crop, with a cultivation area similar to other vegetable crops such as garlic, carrot, and eggplant [1]. Asparagus is a dioecious species with female and male individuals. Male plants are preferred for agricultural production because they have higher yields, have greater longevity than females, and are more tolerant to diseases [2,3]. Most cultivars of the species derive from the Dutch population ‘Violet Dutch’, and as a consequence, they have a narrow genetic base [4,5]. Over the past decade, our laboratory has been working toward increasing the genetic diversity of the cultivated species using wild relatives and the Spanish tetraploid landrace ‘Morado de Huétor’ through a combination of agronomic and genetic approaches [6,7,8,9]. In our breeding program, we are also evaluating several populations and germplasm collections for traits of agronomic importance, such as early yield, spear color, spear size, spear quality, tight heads, sex determination, or disease resistances.

Asparagus is susceptible to a variety of diseases, which can affect potential yield and reduce the spear quality and lifespan of production fields [10]. Currently, most diseases are mainly controlled using cultural practices and chemical fungicides. It is generally accepted that genetic resistance is the most effective and sustainable method of controlling diseases. Screening studies have identified resistant germplasm [11,12,13], but the molecular mechanisms underlying that resistance remain unknown. The development of new asparagus varieties with disease resistance has become a priority, especially in the context of sustainable crop production [14]. 

The most predominant class of resistance (*R*) genes contains nucleotide binding site and leucine-rich repeat (NBS-LRR) domains. The NBS-LRR proteins are historically divided into two subfamilies, Toll/Interleukin-1 receptor (TIR)-NBS-LRR (TNL) and non-TNL-NBS-LRR (nTNL), based on their N-terminal features [15]. The TNL subfamily is abundant in dicot species, but is extremely rare or completely absent in monocots, which suggests that this class is specific for dicotyledons [16,17]. The presence of *TNL* genes in basal angiosperms points out the monocot lineage lost its *TNL* genes near the dicot/monocot split [15]. High-quality reference genome assemblies allow the detailed study of gene structure, function, and evolution. Thus, NBS-encoding genes have been surveyed genome-wide in many sequenced genomes of flowering plants, including the monocots *Oryza sativa*, *Zea mays*, *Sorghum bicolor*, and *Brachypodium distachyon* [18,19,20,21]. 

In the past, asparagus breeding has been hindered by the lack of molecular breeding tools. The recent releases of the high-quality reference genome [22] will facilitate further genetic and genomic studies, and asparagus breeding programs will rapidly benefit from those resources. Identification of candidate genes involved in disease resistance would be a major step for the development of resistant varieties using marker-assisted selection. In this study, we provide comprehensive information on the genomic structures, chromosomal locations, sequence homology, evolutionary duplication history, *cis*-regulatory elements, and expression profiles of 49 NBS-encoding genes in garden asparagus.

## 2. Materials and Methods 

### 2.1. Retrieval and Classification of Asparagus NBS-Encoding Genes 

The complete set of NBS-encoding genes was identified in the asparagus genome using a reiterative process. Firstly, the NBS protein sequences of the closely related *Allium sativum* [23] were used as queries in BLASTP searches against the *Asparagus officinalis* genome (Aspof.V1; Genbank accession GCA_001876935.1) to identify the corresponding members in asparagus. Only the top match of every search was considered. These sequences were in turn used as queries against the asparagus genome to identify new candidates using a cutoff of 30% identity, 30% query coverage, and *E*-value of < 1 × 10^−30^. The NBS domain of the new candidates was confirmed by using the National Center for biotechnology Information (NCBI) Conserved Domain Database (CDD, https://www.ncbi.nlm.nih.gov/cdd/) and E-value of 0.01 [24]. Next, each of these new sequences was used as a query to search the entire genome again to identify additional members. We exhausted the BLAST search until we could not retrieve any new sequences. Then, each of these sequences was surveyed to determine whether they encoded TIR, coiled-coil (CC), or LRR motifs using the Pfam database (http://pfam.xfam.org/), SMART protein motif analysis (http://smart.embl-heidelberg.de/; [25]), and COILS program to detect CC domains with a threshold of 0.9 (http://toolkit.tuebingen.mpg.de/pcoils; [26]). For the RPW8 HMM search, we used the PfamScan tool within the EMBL-EBI bioinformatics framework [27]. Detailed information obtained on protein motifs, domains, and families was used to classify these NBS-encoding genes. In order to complete the identification process, we also used 164 proteins from the monocot *Dendrobium catenatum* downloaded from NCBI using “disease resistance” as the keyword (protein database accessed 20 February 2018), and repeated the search procedure described above. This search resulted in 10 new asparagus sequences. Proteins with no match to NBS proteins (BLASTP search against the NCBI *nr* database) or that were identical to previously identified proteins were excluded from further analysis, and the candidate list was eventually narrowed to 68 annotated *NBS* genes. Using one gene model per locus, we identified 49 nonredundant *NBS* genes. Information on chromosomal location, locus ID, amino acid length, molecular weight, and number of exons was retrieved from the NCBI’s database RefSeq, using the refseqR package [28].

### 2.2. Conserved Domains, Sequence Alignment, and Phylogenetic Analyses

The predicted amino acid sequences of the *NBS* genes were subjected to motif analysis under the Multiple Expectation Maximization for Motif Elicitation system (MEME Suite 4.12.0; http://meme-suite.org) under the following conditions: (1) the optimum motif width was set to 20 and 25; (2) the maximum number of motifs was designed to identify a number of motifs with an *E*-value of < 1 × 10^−10^. For phylogenetic analysis, the alignments of the deduced amino acid sequences from the conserved NBS domain (from Ploop to MHDV) were performed using the MUSCLE program as implemented in the Molecular Evolutionary Genetics Analysis software MEGA version 6 with default options [29]. Eight sequences with a short NBS domain (<200 aa) were eliminated from the matrix because they interfered with a fine alignment. Thus, a total of 41 sequences were finally used to reconstruct the NBS phylogeny in asparagus. The phylogenetic tree was constructed by using the maximum likelihood method based on the JTT matrix-based model implemented in MEGA. All positions containing gaps and missing data were eliminated. The reliability of the interior nodes was assessed using 1000 bootstrap replicates.

### 2.3. Distribution, Cluster Analysis, and Gene Duplications

To define a gene cluster, the following parameters were established: a cluster must contain at least two genes, the distance between two neighboring *NBS-LRR* genes should be <200 kb, and no more than eight genes should be present between the neighboring *NBS-LRR* genes [30]. The predicted full-length coding sequences (CDSs) were used to infer the information of gene duplications. Genes were grouped into gene families according to the criteria defined by [19]: (1) the alignment covered >70% of the longer gene; (2) the aligned region had an identity of >70%. Coverage and identity values were obtained by BLAST searches of all the predicted CDSs against each other. Tandem duplicated genes are defined as those closely related in the same family and clustered together. To investigate the segmental duplication events, 30 genes on the same genomic region, including the 15 flanking genes on each side of a given NBS-encoding gene, were compared by pairwise BLAST analysis between two independent segmental blocks. If more than five gene pairs with synthetic relationships (*E*-value < 1 × 10^−10^) were detected, the two blocks were defined as segmentally duplicated regions [31]. Temporal difference in NBS genes’ expansion was inferred by estimating the proportion of multigene families across 80–90% similarities/coverage thresholds [32]. The Circoletto tool was used to plot sequence similarity [33]. 

### 2.4. Digital Expression Analysis

The full-length CDS coding sequences of the *NBS* genes were employed to query the NCBI Sequence Read Archive (SRA) database (https://www.ncbi.nlm.nih.gov/sra). As of this writing, no asparagus transcriptome has been reported under pathogen infection. To asses which *NBS* gene from this study has expression support under different tissues, we selected two libraries constructed from leaf and root samples from two *Asparagus* spp. Thus, asparagus expression data was obtained through searches against nearly 20 Gb of Illumina reads from the libraries SRX1868868 and SRX1910055 (*A. asparagoides* leaf transcriptome and *A. officinalis* root transcriptome, respectively) using Magic-BLAST as the mapper. The searching parameters were: only one read per hit was counted, length reads were >100 bp, and identity was >90%. Normalized counts of hits were performed using customized scripts to quantify the expression of transcripts from public datasets (https://github.com/NCBI-Hackathons/SimpleGeneExpression).

### 2.5. Identification and Analysis of the Promoter Regions

The assembled sequence of the full asparagus genome (Aspof.V1) was obtained using the BSgenome.Aofficinalis.NCBI.V1 package v.1.0.0 [34] to obtain the promoter sequence (~1500 bp from the ATG start codon) for each predicted NBS-encoding gene. Then, the retrieved sequences were screened against a collection of 27,457 *Arabidopsis* promoters (*Arabidopsis* Promoter Element Discovery Tools, http://stan.cropsci.uiuc.edu/tools.php). *Cis*-regulatory elements (CREs) overrepresented in our dataset (ATHB5 and CBF1) were selected for further analysis. Occurrence and distribution analyses of CREs over a given promoter were performed using Python scripts. The expected frequency of each motif was calculated using the average G+C content of 35% observed in the promoters dataset. Probabilities were estimated based on control sets (2000 Monte Carlo simulations, each set *n* = 43 and 1500 bp length). Other characterized CREs included: two pathogen/elicitor response elements (WBBOXPCWRKY1 [TTTGACY], RAV1-B [CACCTG]); two CREs commonly found in defensin promoters (GT1GMSCAM4 [GAAAAA], RAV1AAT [CAACA]); three elements related to abscisic acid responsiveness, dehydration, and low temperature (MYCATERD1 [CATGTG], MYCATERD22 [CACATG], ABRE [ACGTGTC]); and the sucrose responsive element associated with abiotic stress, SURE2STPAT21 [AATACAAAA] [35,36,37,38].

## 3. Results

### 3.1. Identification of NBS-Encoding Genes

We identified a total of 68 *NBS* genes in the Aspof.V1 genome assembly using the search criteria explained in the Materials and Methods. Using one gene model per locus, a total of 49 asparagus *NBS* genes were extracted and named according to their locations from top to bottom on the chromosomes (from Chr. 1 to Chr. 10). Computational survey of the alternative transcripts revealed that at least 14% of the gene family members display alternative splicing (Table S1). One *NBS* gene has evidence of two alternative variants, three genes of three variants, one gene of five variants, and one gene shows up to seven different alternative transcripts (AoNBS32). Based on the protein domain combinations, the *NBS* genes were grouped into seven classes (Table 1). Among the 49 genes, we identified 42 complete genes that carried both the NBS and LRR domains and 7 partial genes that carried the NBS domain but lacked the N-terminal region, the LRR domain, or both. Approximately 36,760 protein-encoding genes were estimated in the genome assembly. Thus, the *NBS* genes accounted for approximately 0.2% of the predicted protein-encoding genes. The average number of exons detected in the *NBS-LRR* genes was 2.95, which is lower than the estimate (5 exons) in all predicted asparagus genes. 

The TIR domain was not identified in any of the predicted proteins. Thus, the majority of the sequences were classified as non-TIR-LRR. The presence of the CC domain is commonly found in the N terminus of the nTNL proteins. We found strong evidence for CC domains in 35 out of the 49 NBS asparagus proteins (including 29 CNL, 4 RNL, and 2 CN). At least half of the amino acid positions contributing to the predicted CC domains in these 35 proteins were positioned from ~35–60 amino acids (aa) from the N terminus, whereas positions ~35–95 aa from the N terminus represented 73% of the positions in the predicted CC domain. Using the MEME suite for motif-based analysis, we identified one distinct motif, spanning mostly aa positions 30–65: M-[KN]-D-D-L-[QEK]-R-R-[KNQ]-[DR]-A-L-P-R-I-[QK]-[AH]-V-[VL]-[ENH]-[AMD]-A-E-[SR]-[GK][-QR]-[QGI]-[EQ]-[IS]-[TE], which was coincident with the CC pattern identified by the COILS software. A second common signature was also present in 27 sequences in roughly positions 65–95 aa, suggesting that these two expressions may confer the CC domain in asparagus (Figure S1).

### 3.2. Distribution of NBS-Encoding Genes

**Figure 1:**
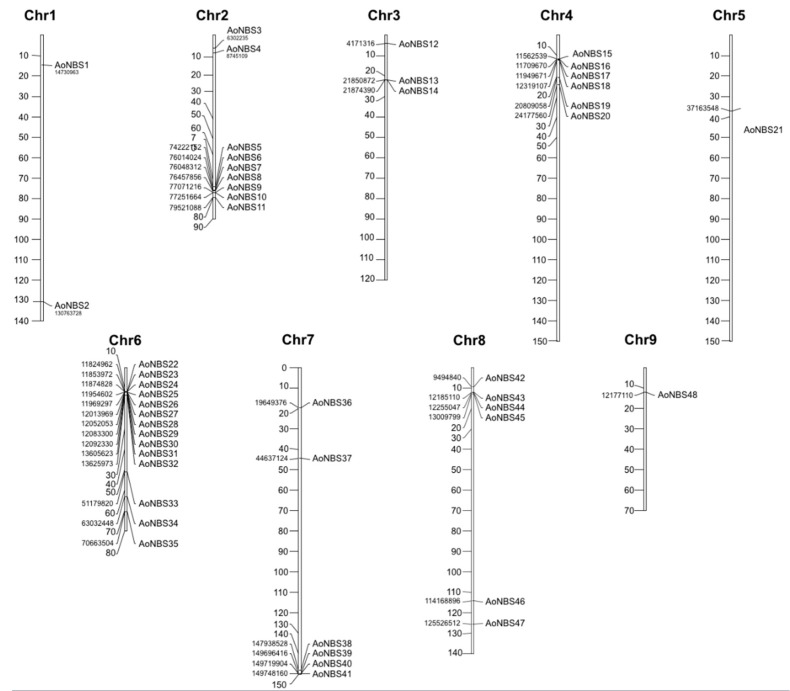
Chromosomal distribution of nucleotide binding sites (NBS) genes on the asparagus genome. Only those chromosomes bearing NBS genes are represented. The chromosome numbers are indicated at the top of each bar. The scale on the left of each chromosome is in megabases (Mb), whereas the coordinates for each locus are shown in base pairs (bp).

The physical locations of the *NBS* genes were determined based on the asparagus gene annotation and the GFF3 file. Using Aspof.V1 genome assembly, we were able to place 48 genes on 9 out of the 10 asparagus chromosomes and 1 gene on the unanchored scaffold. The chromosomal location of the *NBS* genes revealed the uneven distribution of the genes on the asparagus chromosomes and showed tandemly located gene clusters (Figure 1). Chromosome 6 has the highest number (14) of the *NBS-LRR* genes (29% of mapped genes), while chromosomes 5 and 9 have the lowest number (1 each) of the NBS-LRR genes. This pattern of distribution is interesting, because we did not detect association between the chromosome length and the *NBS* loci distribution. We tested this hypothesis through a chi-squared test and we had to reject the null hypothesis; that is, the hypothesis that the *NBS* loci are distributed all over the chromosomes at the same rate based on their length, with a high degree of confidence (*p* = 2.65 × 10^−7^). Chromosome 5 is underrepresented (with just 1 locus), while chromosome 6 contains more loci (14 loci) than expected by chance based on its length (Figure S2). At least one gene containing the CC domain (CNL or RNL) was present on each chromosome. Concerning the cluster of *NBS* genes, out of the 48 mapped genes, 24 genes were present in 9 clusters, each carrying 2 to 3 genes, while another 24 genes were present as singletons (Table 2). Among the 9 clusters, 4 were monophyletic clusters containing 8 genes and 5 were in mixed clusters containing 16 genes. The largest number of genes per cluster (5) was found in the mixed cluster on chromosome 6.

### 3.3. Sequence Alignment and Phylogenetic Analyses

**Figure 2:**
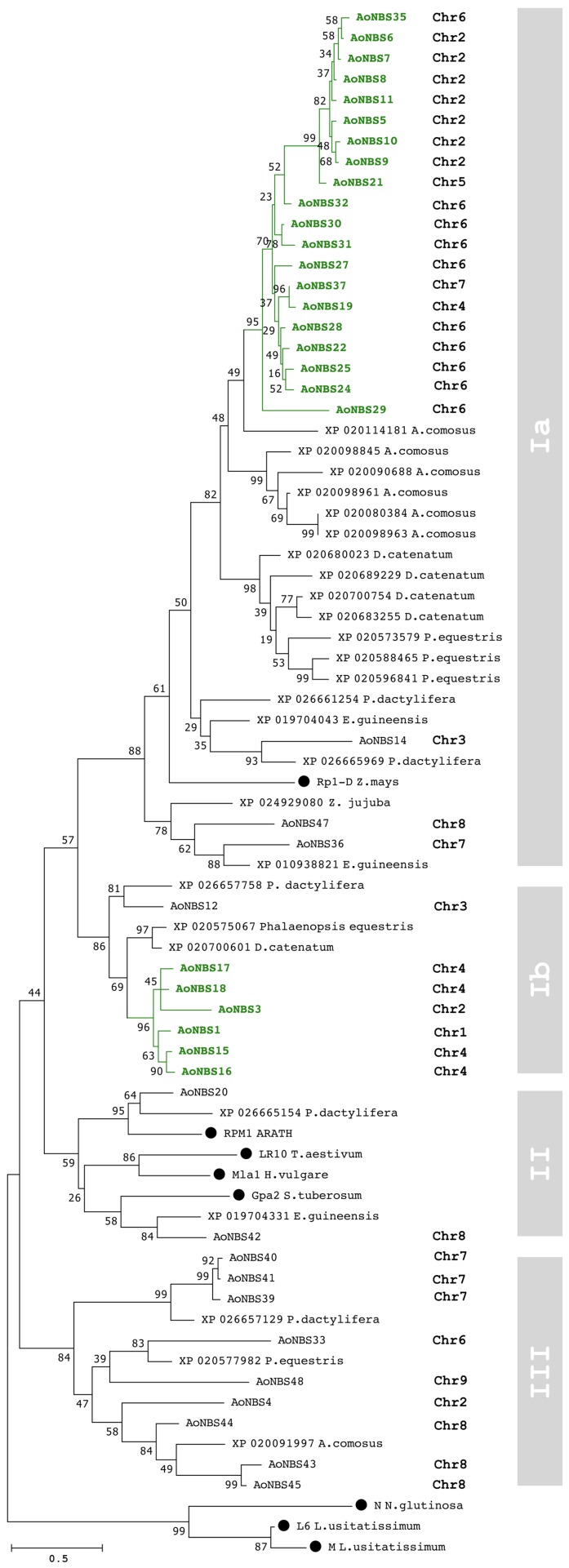
Phylogenetic relationships in asparagus. Maximum likelihood tree based on the NBS domain. Forty-one A*oNBS* were classified into four subclasses. The known resistance (*R)*-genes used for construction of the tree are marked with solid black circles. Their accession numbers are: Mla1 (AAG37356), Gpa2 (AAF04603), Lr10 (AAQ01784), RPM1 (Q39214), Rp1-D (AAD47197), N (AAA50763), L6 (AAA91022), and M (AAB47618). Scale bar represents the number of substitutions per site. Asparagus-specific clades are shown in green color.

To examine the evolutionary relationships among the asparagus *NBS* and known *R*-genes from other plant species, a phylogenetic tree was constructed. None of the asparagus sequences clustered with the three *TNL* genes, namely *N* from *Nicotiana tabacum* and *L6* and *M* from *Linium usitatissimum*. The resulting tree divided the NBS into three groups, AoNBS I to AoNBS III (Figure 2). Group I was further subdivided into Ia and Ib. Each group contained different numbers of AoNBS members, with AoNBS Ia being the largest group with 23 AoNBS proteins, suggesting a large scale of gene duplication events. The genomic distribution of each group followed a particular pattern. Sequences from group Ia are mostly located on chromosomes 2 and 6, and those from group Ib on chromosome 4, whereas most sequences from group III are located on chromosomes 7 and 8 (Figure S3). Percentage amino acid identity per site was derived through (i) pairwise comparison between isolated all NBS groups, (ii) within AoNBS groups, and (iii) between AoNBS groups. When compared with *R*-genes, the identity at the amino acid level ranged from 25% (between AoNBS III and *Rp1*, *LR10*, and *Gpa2* from *Z. mays*, *T. aestivum*, and *S. tuberosum*, respectively) to 43% (between AoNBS II and *RPM1* from *Z. mays*). The amino acid identity within groups ranged from 41% (NBS III) to 76% (NBS IIb), whereas that for between-group comparisons ranged from 26% (NBS Ia and NBS III) to 37% (NBS Ib and NBS II). This pattern suggests a low degree of sequence divergence among asparagus gene clusters, but a relatively high divergence among clusters. Clade NBS III seems to be the most distant from the others. The main group in clade NBSIa also contained sequences from two Orchidaceae (from the same order as asparagus—Asparagales): *Phalaenopsis equestris* and *Dendrobium catenatum,* and the more distantly related species *Ananas comosus*. Clade NBS II clusters AoNBS20 and AoNBS42 with no other asparagus proteins. Interestingly, this clade also contains the *R*-genes *Mla1* from *H. vulgare*, *Gpa2* from *S. tuberosum*, *Lr10* from *T. aestivum*, and *RPM1* from *Arabidopsis*, as well as disease-resistance-like proteins from the monocots *E. guineensis* (XP_019704331) and *P. dactylifera* (XP_026665154), implying that this clade was derived through long-term evolution for conserved functions across plant species. 

### 3.4. Gene Duplications

**Figure 3:**
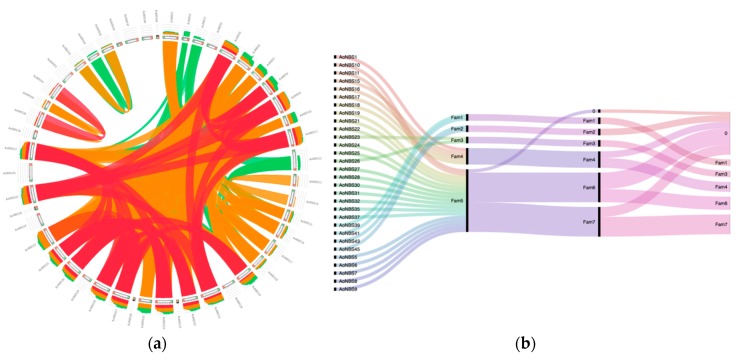
Gene duplications. (**a**) Similarity of NBS genes. Red color shows the highest similarity (>99% identity), followed by orange (95–99%) and green (90–95%) colors. (**b**) Alluvial diagram of the organization in NBS gene families across 70–80–90% similarities/coverage thresholds.

We estimated the proportion of multigene families and analyzed gene duplications to give some insights into the asparagus *NBS* genes’ expansion. The orthologous relationships are shown in Figure 3. The results revealed that approximately two-thirds of all NBS-encoding genes (61.2%) were grouped into 5 multigene families (Table S2). One family comprised ~63% of the gene members (Figure 3b). The maximum number of family members was 19, and the average number of family members was 6. Chromosomes with more than one *NBS* locus are candidates to have undergone local gene duplications. We found a high proportion of *NBS* genes (20 out of 30 duplicated genes) present in 7 clusters (Table 2). On the other hand, a number of genes (10 out of 30) were segmentally duplicated. To distinguish more recent duplications, we used a more stringent criterion for multigene family definition (coverage and identity of 80%, compared to 70%). In this case, the proportion of multigenes among all NBS-encoding genes essentially stayed the same in asparagus (59.2%). We further repeated the analysis to investigate the similarity among closely related genes (coverage and identity 90%, meaning <10% divergence), and we could still place 34.7% of asparagus genes into multigene families (Table S3). More robust analysis of recent duplications was carried out by comparing the mechanisms contributing to their evolution. The proportions of both segmental and tandem duplications decreased using the 90% threshold. However, the segmentally duplicated genes showed a small decrease (20% for segmental genes vs 55% for tandem genes). 

### 3.5. Digital Expression Profiling of the NBS-LRR Genes

To gain an insight into the putative expression patterns of the asparagus *NBS* genes, we studied their profiles by mining two publicly available expression datasets made from root and leaf samples. Overall, the expression analysis indicated that different members are somewhat tissue-specific in expression. We identified a set of *NBS* with larger count numbers in roots from *A. officinalis*. These genes grouped in the phylogenetic clade Ia.

**Figure 4:**
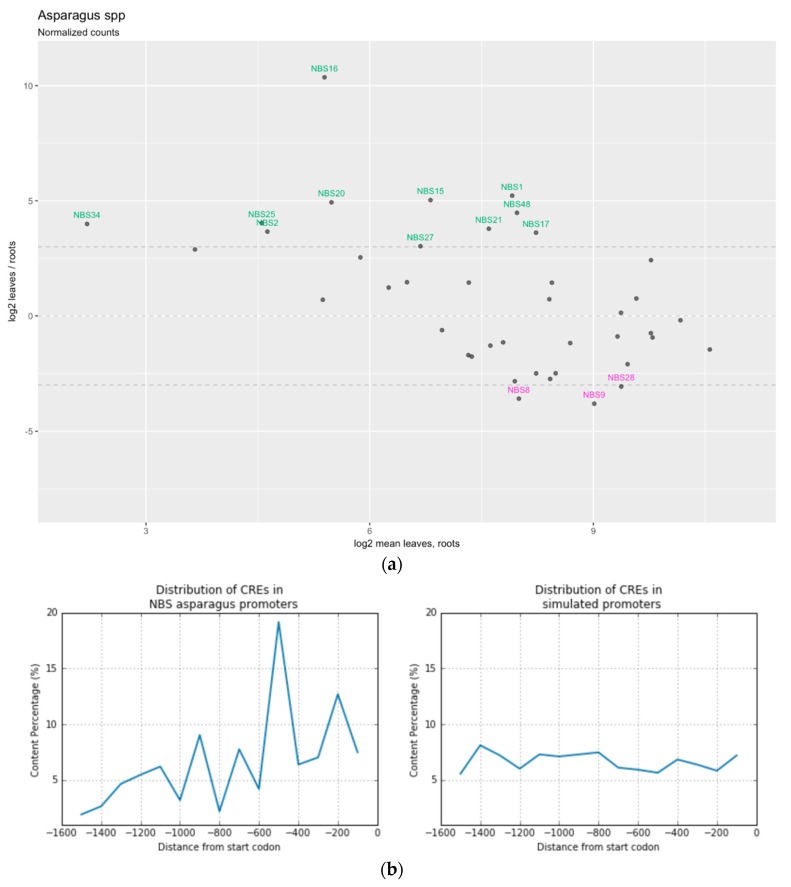
Gene expression analysis in *NBS* genes. (**a**) Expression MA-plot of mean expression signal vs log2-normalized counts of *NBS* genes in two Sequence Read Archive (SRA) libraries. Genes highly enriched (counts ratio >8) in leaf tissues are shown in blue color, whereas NBS enriched in root samples are shown in green color. (**b**) Distribution of *cis*-regulatory elements in NBS promoters. Actual data set vs simulated data set. CREs: *cis*-regulatory elements.

On the other hand, a second group matched with abundance in leaves from *A. asparagoides*. These genes mapped all over the genome chromosomes and clustered across all four clades in the phylogenetic tree. With the exception of A*oNBS18*, the rest of the duplicated members in family 4 (Figure 4a) shared an identical matching pattern (preferentially expressed in leaves). The gene *NBS20*, which is closely related to *R*-genes in the phylogenetic tree, was one of the hits that had the highest number of counts in leaves. This *NBS20* is an obvious candidate for further functional validation.

### 3.6. Promoter Sequence Analysis

In order to give some insight into the dynamic regulation of the *NBS* genes, we screened the proximal and distal regions of promoters (up to 1500 bp upstream of the transcriptional initiation site, TSS) to identify the occurrence and distribution of candidate *cis*-elements that might contribute to the fine regulation of gene expression at the transcriptional level.

We were able to retrieve the promoter genomic sequences from the 48 *NBS* genes that were mapped onto chromosomes. To analyze these regions, first, the 1.5 kb of sequence upstream of the TSS were used to query the GenBank database (*nr*) by BLASTx. The results showed some potential hits for 5 sequences (AoNBS25, AoNBS32, AoNBS33, AoNBS41, and AoNBS46). The genome browser from the NCBI (Genome Data Viewer) confirmed a partial overlap between those sequences and some 5’ or 3’ regions from other annotated asparagus genes. According to the BLAST search, the rest of the 43 surveyed sequences were not coding sequences. Thus, we kept a confident promoter dataset of 43 genomic regions. In a first attempt to analyze the dataset, we screened the sequences against a collection of 27,457 *Arabidopsis* promoters. Two CREs, implicated in either response to pathogens or stress, were overrepresented (AtHB5 and CBF1). Further statistical analysis confirmed an enriched content of those CREs in our asparagus dataset, as well as other CREs associated with plant stress described in the literature (Table 3). To estimate the expected frequency of each CRE, we used the average G+C content of 35% observed in the asparagus dataset (ranging from 33.5 to 36.9). The element RAV1AAT, which is commonly found in defensin promoters, appeared a total of 94 times in 36 sequences (on average, 2.6 elements/promoter). On the other hand, the CBF1/CRT element was present in only three promoters (*N* = 3). However, this CRE was also clearly overrepresented in our query set.

Next, we tested whether any given CRE is more common in certain promoter regions compared to the background. We would not expect enriched elements to show random positions. For this, the promoter sequences were divided into 100-bp nucleotide fragments and the content of the CREs enriched in our dataset was calculated. The control sets based on Monte Carlo simulations produced CREs with a uniform distribution in the promoter region, whereas the real biological sequences showed a positional bias toward the ~−600 to −500 nucleotides upstream from the TSS (Figure 4b).

## 4. Discussion

In this study, we identified 68 NBS-encoding genes that constitute ~0.2% of the total predicted proteins in the asparagus genome. That frequency falls below the range previously observed for other species (0.6–1.76%) [39]. The number of NBS-encoding genes in plant genomes varies substantially, ranging from <100 to >1000 genes [40]. In general, the number of *NBS-LRR* genes is correlated with the total number of genes in the genome [41]. The relationship between NBS-encoding genes and estimated number of genes may indicate that the *NBS* gene family expands neutrally with genome size. However, there are numerous exceptions, such as the quite low copy number of the genomes *Cucumis sativus* (~0.21%, [42]), *Carica papaya* (~0.21%, [43]), or *Citrullus lanatus* (0.19%, [31]), to name a few examples. The asparagus *NBS* gene frequency reveals similar abundance with those species labeled as having genomes with a small *NBS* gene family. Motivated by the weak relationship between *NBS-LRR* gene frequency and total number of annotated genes, several studies have shown that the seemingly low *NBS* gene count in some species is best described by a nonlinear correlation, so that number of *NBS* genes evolved among plant species regardless of genome size [44,45]. In this scenario, the seemingly low *NBS* gene count in some species is relative, as each plant genome may have the minimum number of NBS proteins required for adequate surveillance [39]. The substantial variation in the size of different NBS-LRR families across species that we observe may be the result of the independent expansion of different NBS-LRR families among plant species [40]. The question that remains then is what biological mechanism(s) define the great variability in *NBS* gene abundance that we observe among plant species. Plant biologists are currently debating over at least four different hypotheses to explain the seemingly low copy number of *NBS-LRR* genes. It is important to note that not all of these different scenarios are mutually exclusive. On the contrary, some of the following hypotheses may function together: (i) *Fitness cost*. One explanation might be due to the fitness cost and lethal effect of *R*-genes on plant genomes [40]. Since defense is energetically expensive, plants are faced with the dilemma of allocating valuable resources to growth or to defense. In situations without any pathogens, plants that are less defended should be fitter than those that are more defended. The same adverse effects are often observed in transgenic plant lines overexpressing *NBS-LRR* genes [46]; (ii) *Host–pathogen coevolution*. Positive selection drives host–pathogen coevolution and selection for new resistance [47]. The NBS expansion is presumably driven by the distinct pathogen pressure faced by each species. If few pathogens have evolved to adapt to specific genomes, the *NBS-LRR* genes are not under strong selection pressure [48,49]; (iii) *Indirect detection*. The ‘guard model’ proposes that a resistance protein may maximize its detection potential by monitoring a host protein targeted by multiple pathogen effectors. Therefore, it is theorized that few *R*-genes are capable of targeting the broad diversity of pathogens in plants [50]. This hypothesis suggests that some *NBS-LRR* genes could be preserved (molecular integrity or conserved function) for an extremely long period of time [51]; (iv) *Complementary mechanisms*. The apparent deficiency of *NBS-LRR* genes may be compensated by a diverse group of other gene classes that actively contribute to plant defense. The notable expansion of the lipoxygenase (*LOX*) genes in the cucumber and watermelon genomes has been suggested as a defense mechanism beyond NBS-encoding genes [31,52,53]. As more gene families become characterized and available in asparagus, a clearer picture of the possible relationship between NBS and other defense categories will emerge. From a breeding perspective, it would be of interest to further explore this hypothesis.

Despite the relatively low number of NBS-encoding genes, the asparagus genome contains the main NBS groups and subgroups observed in other plant species. As expected, we did not detect NBS-encoding genes belonging to TNL/TN subgroups. These results are congruent with other monocots [20,32,54]. The presence of TNL genes in early-diverging angiosperms suggests that these genes were most likely also present in the ancestor of angiosperms, but were independently lost in monocot lineages [15]. In addition, the AoNBS proteins contain all necessary structural motifs to function as known NBS proteins. BLAST searches comparisons showed that AoNBS-encoding genes possess high similarity to other *NBS* genes from monocots.

Up to 26 asparagus NBS predicted proteins (from 7 loci) show evidence of alternative variants. Multiple reports have linked alternative splicing to the activation of plant immune responses. One of the first studies on alternative splicing in NBS regulation showed the critical importance of two tobacco isoforms of a *TNL* gene to confer full resistance against the tobacco mosaic virus [55]. Since then, many cases of the regulation of resistance genes have been associated to TNL, with recent examples reported for *CNL* genes in monocots [56,57]. Although the biological significance is not understood in great detail at this point, the alternative splicing of *NBS-LRR* genes may facilitate the genetic evolution of resistance by creating a new function that is distinct from the original protein [46,58]. In this way, alternative splicing in *NBS* gene regulation may represent an additional mechanism underlying diversity in NBS proteins. It would be of interest to characterize the functional roles of some asparagus isoforms. Uncovering the function of these alternative variants would be a major step toward a deeper understanding of plant–pathogen coevolution.

The main factor driving the expansion of the *NBS* gene family in both monocot and eudicot lineages is gene duplication. The genomic dispersion of the A*oNBS* sequences suggests a number of local duplication events (clade Ia, Figure 2) and/or the duplication of a small group of genes to an unlinked locus (segmental duplications). It is interesting that the asparagus genome shows increased duplications, measured as a percentage of multigenes, when compared to *Arabidopsis* or the genomes of the monocots rice and *Brachypodium distachon*. However, we did not observe an increased number of families. This seems to indicate that the expansion of *NBS* genes through gene duplication is mainly caused by an increase in the number of genes within a group, and not by an increase in different groups. The high proportion of sequences present in clusters (Table 2) suggests that tandem duplication could have played a major role in the expansion of NBS-encoding genes. Using the stringent criteria—coverage and identity of 90%—for multigene family definition, the asparagus genome shows one of the largest proportions of genes grouped into families when compared to other species with available data using the same criteria (Table S3). In other words, in the asparagus genome, more genes are detected as being members of families from recent duplication events (N = 17) than those having more ancient origin (N = 30 − 17 = 13), suggesting that the recent expansion of A*oNBS* genes is the prevalent force in gene duplication leading to increased gene numbers. The analysis shows different patterns in the expansion of asparagus *NBS* genes: (i) At early stages of evolution, the mechanism of duplication is driven by gradual expansion of tandemly duplicated genes; (ii) More recent expansion involves both the mechanisms of tandem genes and duplication events across multiple chromosomes. Almost all the segmental duplications that we can detect have evolved from these recent events.

We used public SRA databases to find evidence of the expression of the asparagus *NBS* genes. We mapped the *NBS* sequences against the two libraries using Magic-BLAST, a novel tool allowing the mapping of large next-generation sequencing runs against a reference database [59]. Digital analyses play an increasingly important role for providing gene expression information and facilitating future genomic functional studies of plant growth and development. There is a need for expression analysis and quantitative trait loci (QTL) identification of disease resistances in asparagus. Unfortunately, to date, no study of the asparagus transcriptome has been reported under pathogen infection. However, a few libraries have been developed for some *Asparagus* spp. tissues, and our survey of publicly available libraries allowed us to reach some conclusions. The expression results give support for various functional roles of *NBS* genes, with different members displaying expression preferences to specific tissues. This suggests that asparagus NBS-encoding genes may have spatial expression patterns, which vary by tissue type or genotype (it should be noted that we used two asparagus species). Furthermore, the expression data revealed that most of the duplicated members within a group exhibited the same expression pattern. This suggests that the functional conservation of the duplicated family members is a major feature of the evolution of these genes. Future association studies on different diseases using contrasting genotypes may reveal a role for the genes and clusters that we have detected in this study.

Finally, the analysis of the promoters signifies that CREs most likely play important roles in the activation of the *NBS* gene expression. Most of the sequences contained the element RAV1AAT, which is commonly found in defensin promoters. The RAV1AAT elements regulate the expressions of multiple defense-related genes through the binding of RAV1 transcription factor proteins [60]. The abundance of the RAV1AAT element commonly found in the promoters of *NBS* genes indicates that this element is important for the regulation of defense-related responses [61,62]. Overall, the class of the most abundant motifs found in the asparagus NBS promoters indicates the significant cross-talk between abscisic acid-responsive signal transduction, abiotic stress responses, and disease tolerance-related signaling pathways [63,64,65]. In general, not only the total number of CREs, but also their position may reveal their functional significance. The asparagus promoters showed a positional bias toward the 600–500 nucleotides upstream from the TSS. This clearly needs to be kept in mind in studies on the regulation of gene expression and promoter functionality [38].

## 5. Conclusions

In summary, we applied a number of computational approaches that provided an integrated framework for the analysis and comprehensive cataloging of the *NBS* gene family in the asparagus genome. The *NBS* genes were unevenly distributed through the asparagus genome. Our study may provide an excellent resource for plant breeders aimed at colocalizing disease resistance QTL with NBS-encoding genes. It will be exciting to determine if they match with the significantly enriched hotspot region in chromosome 6. Some asparagus genes are promising targets for further characterization based on their close phylogenetic relationships with some *R*-genes of known function. Our data suggest that the major gene duplication event is driven mostly by a single family with a high number of members. The prevalent force in gene duplications is based on tandem pairs, but segmental duplications have also played a fundamental role in the recent expansion of the *NBS* gene family. Comparative expression analysis between asparagus species indicated that some members displayed preferential expression in leaf tissues. Members from the same duplicated family may perform redundant physiological functions. Our study has practical value in agriculture as it will accelerate future efforts in resistance breeding in this crop. Our study also provides a foundation for further comparative genomic analyses and a framework to trace the dynamic evolution of *NBS* genes on a large timescale within the Asparagales order.

## Figures and Tables

**Table 1:** Number and classification of NBS-encoding genes in the asparagus genome.

Predicted Protein Domain	Letter Code	No. of Genes
**NBS-LRR-type genes**		
TIR-NBS-LRR subclass	TNL	0
non-TIR-NBS-LRR subclass		
CC-NBS-LRR	CNL	29
NBS-LRR	NL	6
RPW8-NBS-LRR	RNL	4
X_CC_-NBS-LRR	XNL	3
**NBS-type genes**		
NBS	N	4
CC-NBS	CN	2
X_CC_-NBS	XN	1
**Total genes**		**49**

TIR: Toll/interleukin-1-receptor, CC: coiled-coil domain, LRR: leucine-rich repeat domain, NBS: nucleotide binding site. CN, TCN, and XN are structurally incomplete genes. NL and XNL are sequences with the N-terminal region comparable in length to an intact CNL, but we could not identify the CC domain based on the COILS program. The CC domain was detected in XNL sequences only by the Conserved Domain database of NCBI.

**Table 2:** Cluster analysis of NBS-LRR domains in asparagus.

Cluster	Cluster Size (kb)	No. of NBS Genes	No. of Genes Neighboring	Chr.	Gene ID	NBS Class
1	31	2	0	2	AoNBS6, AoNBS7	CNL
2	180.45	2	7	2	AoNBS9 AoNBS10	CNL, CN
3	3.43	2	0	3	AoNBS13, AoNBS14	CNL
4	144.67	2	3	4	AoNBS15, AoNBS16	RNL
5	126.09	5	6	6	AoNBS22, AoNBS23, AoNBS24, AoNBS25, AoNBS26	CNL, N
6	66.09	4	2	6	AoNBS27, AoNBS28, AoNBS29, AoNBS30	CNL, NL
7	16.61	2	2	6	AoNBS31, AoNBS32	CNL
8	36.92	3	2	7	AoNBS39, AoNBS40, AoNBS41	CNL, NL
9	60.08	2	1	8	AoNBS43, AoNBS44	NL, CNL

**Table 3:** *Cis*-regulatory elements (CRE) in asparagus NBS promoters (no. Of queries = 43).

CRE	Motif	Promoters Observed	Occurr. Observed	Avg. Occurrs. per Promoter	Occurrs. Expected	Enrichment Factor	*p*-Value
CBF1/CRT	TGGCCGAC	3	3	1	0	∞	0.00150
AtBH5	CAATNATTG	12	15	1.3	3	5	0.00444
SURE1STPAT21	AATAGAAAA	7	7	1	2	3.5	0.00505
ABR	ACGTGTC	6	6	1	2	3	0.00774
RAV1-B	CACCTG	9	12	1.3	6	2	0.01440
MYCATERD22	CACATG	15	23	1.5	12	1.9	0.01559
WBBOXPCWRKY1	TTTGACY	16	19	1.2	11	1.7	0.01408
GT1GM4SCAM4	GAAAAA	30	70	2.3	45	1.6	0.02178
MYCATERD1	CATGTG	12	17	1.4	12	1.4	0.02038
RAV1AAT	CAACA	36	94	2.4	66	1.4	0.02179

Occurrs., number of ocurrences

## Data Availability

The datasets generated during the current study are available in our *Asparagus* repository (https://github.com/jdieramon/AsparagusProject).

## Supplementary Materials

The following are available online at http://www.mdpi.com/2073-4425/9/12/568/s1, Figure S1: Analysis of the N-terminal domain in non-TNL sequences. (a) Regular expression of the 30–65 and 65–95 amino acid regions from asparagus sequences. Sequence logo was generated from multiple alignments using MEME suite. (b) Amino acid position of predicted CC domain., Figure S2: Ratio of NBS loci observed vs expected based on uniform distribution according to chromosome length. Green color denotes ratios >2-fold, whereas magenta color denotes ratios <2-fold, Figure S3: Chromosome distribution over phylogenetic clades, Table S1: NBS gene family in *Asparagus officinalis*. AoNBS49 was not mapped on any chromosome, Table S2: Organization in families of NBS genes in four plant genomes, Table S3: Organization in families of NBS genes in plant genomes. The stringent criterion coverage and identity of 90% was used for multi-gene family definition.
