# Supplementary material for: Segmental and Tandem Duplications Driving the Recent NBS-LRR Gene Expansion in the Asparagus Genome
Source: Genes (Basel). 2018 Nov 23;9(12):568. doi: 10.3390/genes9120568 (PMC6316259; doi:10.3390/genes9120568)
Supplement: Supplementary file 1 [file genes-09-00568-s001.zip › genes-365377-SI.pdf]

Genes 2018, 9, 568; doi:10.3390/genes9120568

Segmental and tandem duplications driving the recent NBS-LRR gene expansion in the asparagus genome

Die, Castro, Millán, Gil

Supplementary Files

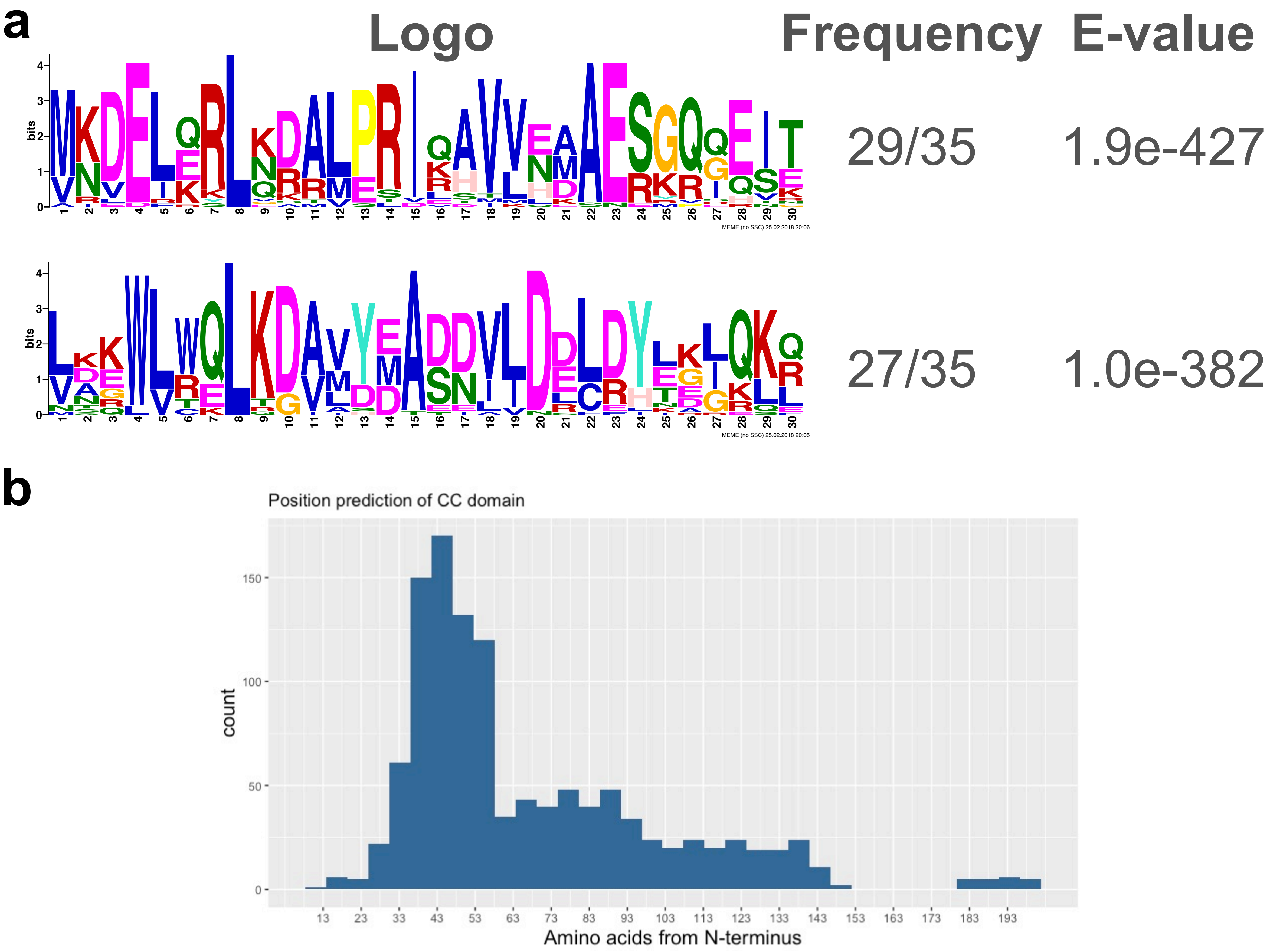

Figure S1: Analysis of the N- terminal domain in non-TNL sequences. (**a**) Regular expression of the 30-65 and 65-95 amino acid regions from asparagus sequences. Sequence logo was generated from multiple alignments using MEME suite. (**b**) Amino acid position of predicted CC domain.

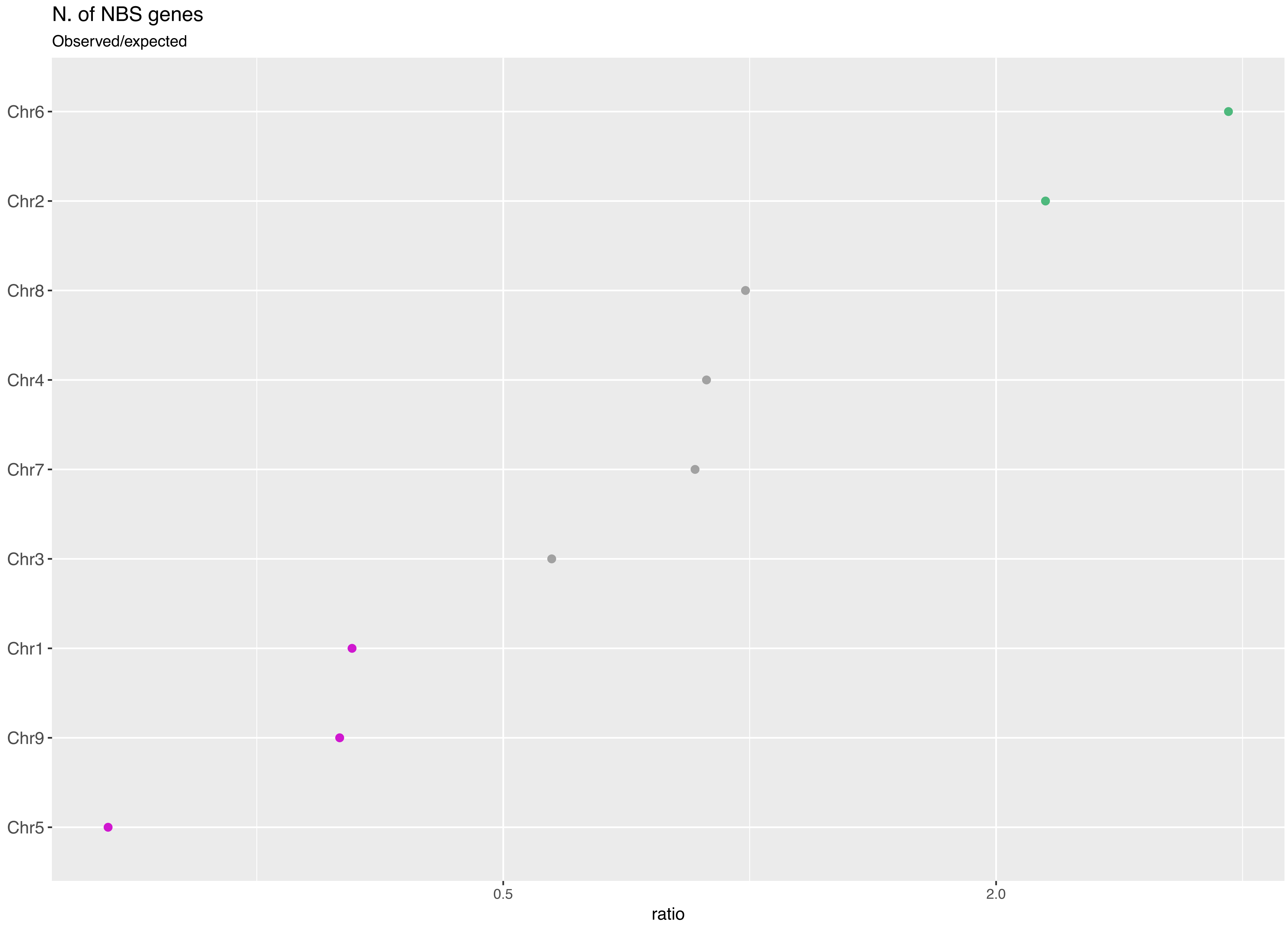

Figure S2: Ratio of NBS loci observed vs expected based on uniform distribution according to chromosome length. Green color denotes ratios >2-fold, whereas magenta color denotes ratios < 2-fold.

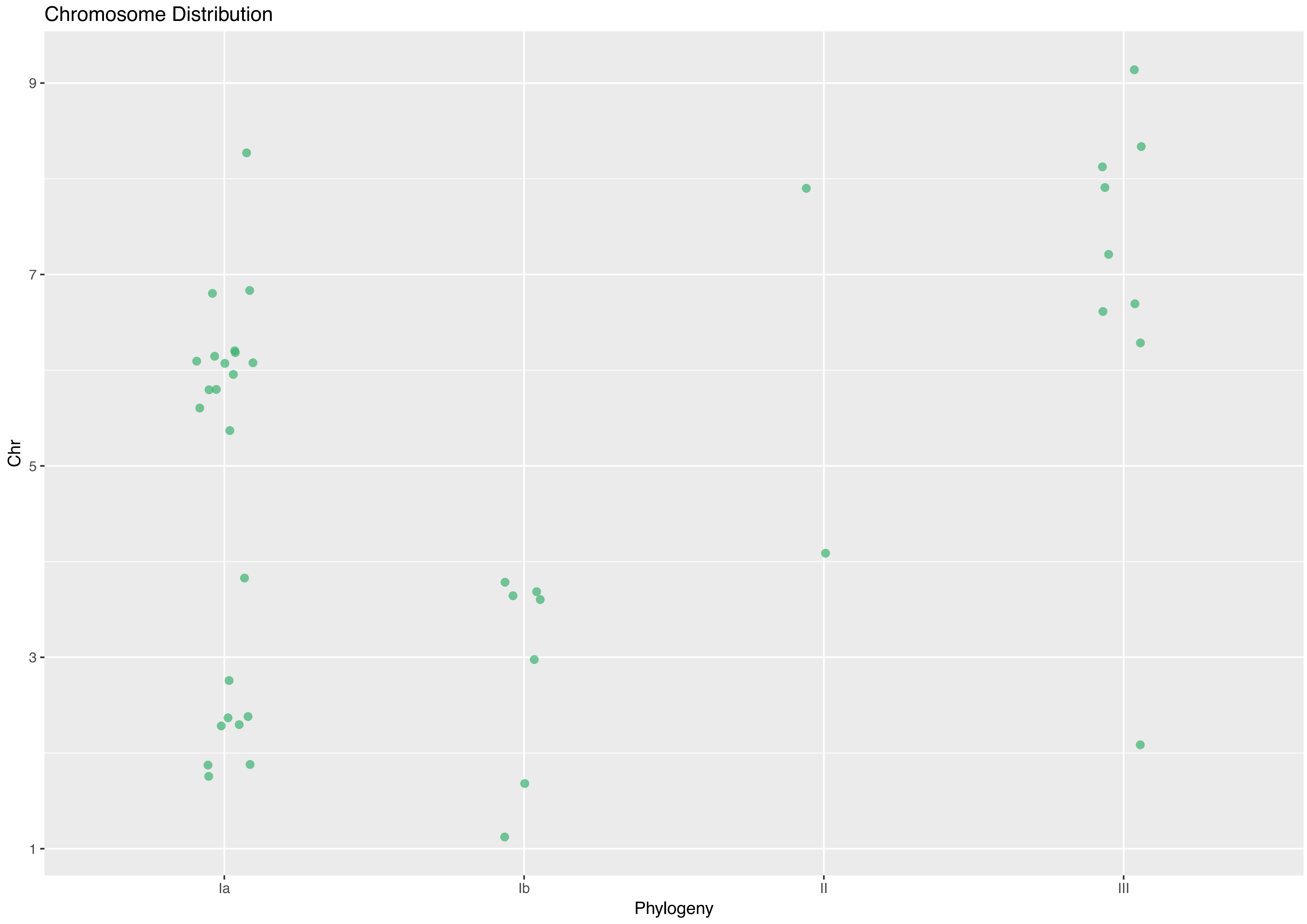

Figure S3: Chromosome distribution over phylogenetic clades.

Table S1. NBS gene family in *Asparagus officinalis*.

GeneID	XP	LOC	Chr	chr_start	chr_end	Strand	AA	mol_wt	exon	N.Isof
AoNBS1	XP_020253886	LOC109830941	1	14730963	14727608	-	1104	124.04	2	1
AoNBS2	XP_020266430	LOC109841914	1	130763725	130767761	+	513	58.39	4	1
AoNBS3	XP_020253701	LOC109830750	2	6302235	6294422	-	781	88.33	4	1
AoNBS4	XP_020253744	LOC109830788	2	8745109	8742884	-	741	85.54	1	1
AoNBS5	XP_020254559	LOC109831614	2	74222152	74218076	-	1210	135.77	1	1
AoNBS6	XP_020254608	LOC109831652	2	76014024	76017316	+	1026	114.74	1	1
AoNBS7	XP_020254254	LOC109831334	2	76048314	76057593	+	824	92.79	3	1
AoNBS8	XP_020254638	LOC109831672	2	76457854	76453803	-	1020	114.53	1	1
AoNBS9	XP_020254648	LOC109831678	2	77071219	77066886	-	1241	139.55	5	3
AoNBS10	XP_020254269	LOC109831350	2	77251666	77254648	+	738	83.68	2	1
AoNBS11	XP_020254735	LOC109831747	2	79521088	79527213	+	1234	138.05	2	5
AoNBS12	XP_020255460	LOC109832521	3	4171316	4164677	-	1456	164.28	7	1
AoNBS13	XP_020256584	LOC109833349	3	21850872	21870961	+	851	84.18	4	1
AoNBS14	XP_020256581	LOC109833347	3	21874390	21884220	+	1195	136.2	2	1
AoNBS15	XP_020262233	LOC109838182	4	11562539	11565004	+	822	92.16	1	1
AoNBS16	XP_020262234	LOC109838184	4	11709670	11713279	+	1015	114.28	3	1
AoNBS17	XP_020262239	LOC109838188	4	11949671	11954877	+	1155	129.25	1	1
AoNBS18	XP_020260201	LOC109836641	4	12319107	12313472	-	1022	98.48	3	3
AoNBS19	XP_020262355	LOC109838315	4	20809058	20805407	-	1151	94.49	3	1
AoNBS20	XP_020262401	LOC109838361	4	24177560	24164226	-	934	107.71	2	1
AoNBS21	XP_020266254	LOC109841718	5	37163549	37170184	+	1404	159.21	5	1
AoNBS22	XP_020270608	LOC109845747	6	11824962	11828662	+	1110	123,443.67	3	1
AoNBS23	XP_020270609	LOC109845748	6	11853972	11854427	+	151	16.23	1	1
AoNBS24	XP_020270610	LOC109845749	6	11874828	11885980	+	1232	138.52	3	1
AoNBS25	XP_020270612	LOC109845752	6	11954602	11957533	+	913	102.84	2	1
AoNBS26	XP_020270614	LOC109845754	6	11969297	11969758	+	153	16.5	1	1
AoNBS27	XP_020270617	LOC109845758	6	12013969	12019211	+	1232	109.22	3	1
AoNBS28	XP_020268733	LOC109844186	6	12052053	12058625	+	1223	137.35	4	1

Table S1. NBS gene family in *Asparagus officinalis* (cont.).

AoNBS29	XP_020270619	LOC109845759	6	12083300	12085923	+	689	16.26	6	1
AoNBS30	XP_020268735	LOC109844187	6	12092330	12098847	+	1172	132.47	4	2
AoNBS31	XP_020269341	LOC109844633	6	13605623	13609362	+	1114	107.38	3	1
AoNBS32	XP_020269333	LOC109844631	6	13625973	13658494	+	1243	139.42	9	7
AoNBS33	XP_020270986	LOC109846174	6	51179819	51174237	-	914	102.65	4	1
AoNBS34	XP_020269898	LOC109845106	6	63032450	63033580	+	264	29.4	1	1
AoNBS35	XP_020271110	LOC109846295	6	70663507	70659375	-	1266	92.75	2	1
AoNBS36	XP_020271621	LOC109846787	7	19649375	19653388	+	1277	144.85	2	1
AoNBS37	XP_020271898	LOC109847062	7	44637125	44633251	-	1221	137.42	3	1
AoNBS38	XP_020272777	LOC109847959	7	147938534	147928209	-	624	74.22	8	1
AoNBS39	XP_020273079	LOC109848139	7	149696416	149704334	+	1093	123.39	5	3
AoNBS40	XP_020272805	LOC109847982	7	149719902	149726796	+	652	73.28	2	1
AoNBS41	XP_020272808	LOC109847984	7	149748154	149766107	+	1153	130.71	5	1
AoNBS42	XP_020244412	LOC109822593	8	9494840	9499026	+	920	104.56	3	3
AoNBS43	XP_020242652	LOC109820867	8	12185110	12158006	-	929	106.78	2	1
AoNBS44	XP_020242654	LOC109820869	8	12255047	12245195	-	848	95.9	3	1
AoNBS45	XP_020244702	LOC109822852	8	13009799	12993300	-	885	101.11	2	1
AoNBS46	XP_020242295	LOC109820548	8	114168900	114170503	+	388	43.14	1	1
AoNBS47	XP_020241782	LOC109820118	8	125526511	125521372	-	922	104.47	3	1
AoNBS48	XP_020245082	LOC109823207	9	12177110	12174714	-	754	84.81	2	1
AoNBS49*	XP_020250123	LOC109827523	Un	0	0	-	186	21.73	3	1

*AoNBS49 was not mapped on any chromosome.

Table S2. Organization in families of NBS genes in four plant genomes.

	Asparagus	*B. distachon*	Rice	*Arabidopsis*
Single-genes	19	77	216	93
Multi-genes	30	49	248	81
Gene families	5	20	93	25
Max. family members	19	7	10	7
Avg. family members	6	2.45	2.67	3.24
Multi-genes/single genes	1.58	0.64	1.15	0.87
% Multi-gene families	61.2%	38.9%	53.4%	46.6%

Data for *B. distachon*, rice and *Arabidopsis* taken from Tan and Wu Comp. Funct. Genomics 2012, 418208 (2012).

Table S3. Organization in families of NBS genes in plant genomes. The stringent criterion coverage and identity of 90% was used for multi-gene family definition.

	Asparagus	*F. vesca*	*M. domestica*	*P. breschneideri*	*P. persica*	*P. mume*	*C. mollissima*
Single-genes	32	128	565	298	244	270	418
Multi-genes	17	16	183	171	110	82	101
Gene families	5	5	62	66	35	30	41
Max. family members	6	5	9	10	8	7	5
Avg. family members	3.4	3.20	2.95	2.59	3.14	2.73	2.46
% Multi-gene families	34.69	11.11	24.47	36.46	31.07	23.30	19.46

Data for *F. vesca*, *M. domestica*, *P. bretschneideri*, *P. persica* and *P. mume* taken from Yang et al. Mol. Genet. Genomics 280, 187-198 (2008).
Data for *C. mollissima* taken from Zhong et al. Sci Rep.5, 16638 (2015).
